# Up-Regulation of p53/miR-628-3p Pathway, a Novel Mechanism of Shikonin on Inhibiting Proliferation and Inducing Apoptosis of A549 and PC-9 Non–Small Cell Lung Cancer Cell Lines

**DOI:** 10.3389/fphar.2021.766165

**Published:** 2021-11-16

**Authors:** Jieli Pan, Meiya Li, Fenglin Yu, Feiye Zhu, Linyan Wang, Dandan Ning, Xiaoli Hou, Fusheng Jiang

**Affiliations:** ^1^ Academy of Chinese Medical Sciences, Zhejiang Chinese Medical University, Hangzhou, China; ^2^ College of Life Science, Zhejiang Chinese Medical University, Hangzhou, China

**Keywords:** shikonin, non–small cell lung cancer, p53, miRNA, proliferation, apoptosis

## Abstract

Shikonin (SHK) is a pleiotropic agent with remarkable cell growth inhibition activity against various cancer types, especially non–small cell lung cancer (NSCLC), but its molecular mechanism is still unclear. Our previous study found that miR-628-3p could inhibit the growth of A549 cells and induce its apoptosis. Bioinformatics analysis predicted that miR-628-3p promoter sequence contained p53 binding sites. Considering the regulatory effect of SHK on p53, we speculate that SHK may inhibit the growth and induce apoptosis of NSCLC cells by up-regulating miR-628-3p. CCK-8 and EdU assay confirmed the inhibitory effect of SHK on A549 and PC-9 cells. Meanwhile, quantitative reverse transcription–polymerase chain reaction and Western blot showed that SHK could promote the expression of p53 and miR-628-3p in a dose-dependent manner. Overexpression of p53 or miR-628-3p can inhibit the growth and promote apoptosis of A549 and PC-9 cells, while silencing p53 or miR-628-3p has the opposite effect. Dual luciferase reporting assay and ChIP (chromatin immunoprecipitation) assay further verified the direct interaction between p53 and the promoter of miR-628-3p. Gene knockdown for p53 or miR-628-3p confirmed that SHK inhibits the growth and induces apoptosis of A549 and PC-9 cells at least partly by up-regulating p53/miR-628-3p signaling pathway. Therefore, these novel findings provide an alternative approach to target p53/miR-628-3p axis and could be used for the development of new treatment strategies for NSCLC.

## Introduction

Lung cancer is the most often diagnosed cancer (approximately 11.6% of all cancer cases) and the leading cause of cancer death worldwide (18.4% of overall cancer mortality) (Bray et al., 2018); it places a heavy burden on the health care system and causes a significant challenge to clinicians and patients. According to histology, lung cancer can be classified into small cell lung cancer (SCLC) and non–small cell lung cancer (NSCLC) (Goldstraw, 2011). NSCLC, a subtype of lung cancer, accounts for approximately 85% of cases, which is the most common cause of cancer death. Currently, surgery, radiation therapy, chemotherapy, and immunotherapy are widely used in clinical practice; especially chemotherapy for NSCLC (such as anaplastic lymphoma kinase inhibitors, tyrosine kinase inhibitors, and epidermal growth factor receptor inhibitors) has shown better clinical outcome than that for SCLC (Wang et al., 2020). These results indicated that the development of small molecule drugs for the treatment of NSCLC is still of great significance.

Shikonin (SHK), a natural naphthoquinone pigment isolated from *Lithospermum erythrorhizon*, has been reported to suppress the growth of various cancer types *in vitro*, such as lung (Kim et al., 2017; Li et al., 2018; Fayez et al., 2020), breast (Du et al., 2020; Wang et al., 2021), skin (Li et al., 2015), gallbladder (Zhai et al., 2017), glioma (Ma et al., 2020), and prostate (Gara et al., 2015). One clinical trial proved that SHK is beneficial for late-stage lung cancer patients, who were not candidates for operation, radiotherapy, or chemotherapy (Guo et al., 1991). Researches on different lung cancer cell lines have shown that SHK can elicit its growth inhibitory activity by inducing apoptosis, necrosis, autophagy, and senescence, and its mechanism involves regulating the multiple signaling pathways such as p53, ERK, STAT3, EGFR, and so on (Eric et al., 2020). However, the exact antitumor molecular mechanism of SHK remains to be elucidated.

MicroRNAs (miRNAs) are small noncoding RNA molecules that inhibit gene expression at the transcriptional and posttranscriptional level by binding to the 3′-untranslated region of the target mRNAs (Wang et al., 2013). In cancer cells, miRNAs can act as carcinogens or tumor suppressors, depending on their target genes and cell types (Kooshkaki et al., 2020). A large number of studies have shown that anticancer drugs can inhibit tumor cell proliferation, epithelial–mesenchymal transition, and drug efflux; induce tumor cell apoptosis and autophagy; and improve the sensitivity of cancer cells to anticancer therapy by interfering with miRNA (Seo et al., 2019; Kooshkaki et al., 2020). Therefore, miRNA can be used as a target for anticancer drug research and development. Recent studies have shown that SHK can inhibit the proliferation of retinoblastoma by up-regulating miR-34a and miR-202 (Su et al., 2018), and it can also suppress progression and epithelial–mesenchymal transition in hepatocellular carcinoma cells by modulating miR-106b (Li and Zeng, 2020). Apparently, regulating miRNA is another potential anticancer molecular mechanism of SHK.

Recent studies have reported that miR-628-3p was down-expressed in breast (Dong et al., 2019), gastric (Chen et al., 2015), and pancreatic cancer (Jiang, 2017) comparing with its adjacent normal tissue, and overexpression miR-628-3p can inhibit cancer cell growth and migration and induce cancer cell apoptosis (Chen et al., 2015; Jiang, 2017; Dong et al., 2019). Consistent with those reports, our previous work demonstrated that up-regulated miR-628-3p can inhibit migration and promote apoptosis in A549 cells by negatively regulating HSP90 (Pan et al., 2018). Further bioinformatics analysis showed that miR-628-3p transcription might be regulated by p53. Meanwhile, several studies have shown that SHK can inhibit the growth and induce apoptosis of A549 cells by promoting the expression of p53 (Yeh et al., 2015; Zheng, 2017; Zheng et al., 2018). Herein, the objective of the current study was to reveal whether SHK can inhibit the proliferation and induce apoptosis of NSCLC cells by regulating the p53/miR-628-3p pathway.

## Materials and Methods

### Cell Culture

293T human NSCLC cell lines A549 and PC-9 were purchased from the Type Culture Collection of the Chinese Academy of Sciences, Shanghai, China. The cells were cultured in Dulbecco modified Eagle medium and RPMI 1640 medium (Invitrogen, United States) supplemented with 10% fetal bovine serum (Gibco, United States) and 1% antibiotics (100 U/ml penicillin and 100 μg/ml streptomycin sulfate) in 5% CO_2_ at 37°C.

### Transfection

miRNA mimics (miR-628-3p mimics, miR-NC), miRNA inhibitors (anti-miR-NC, anti-miR-628-3p), and si-RNAs (si-NC, si-p53) (See Supplementary Table S1 for details) were designed and synthesized by Ribobio company (Guangzhou, China). The p53 overexpression vector (pHY-819-p53) was commercially constructed by Hanyin Company (Shanghai, China) and empty pHY-819 vector (see Supplementary Figure S1) was used as the negative control. All oligonucleotides and plasmids were transfected into 293T, A549, and PC-9 cells using the Lipofectamine 3000 Transfection Reagent (Invitrogen) according to the manufacturer’s instructions.

### CCK-8 Assay

SHK (HPLC ≥98%, B21682, Shanghai Yuanye Bio-Technology Co., Ltd. Shanghai, China) was dissolved in dimethyl sulfoxide (DMSO) and diluted into a series of concentrations with culture medium (the final concentration of DMSO was 0.1%). After cells were treated in triplicate with the various concentrations of SHK or vehicle (medium containing 0.1% DMSO), CCK-8 was added to the cell suspension and incubated for 2 h. The optical density (OD) at 450 nm in each well was measured using a microplate reader (PE enpire; United States). The OD resulting in control cells was defined as 100% cell viability, and all other measurements were expressed as a percentage of the control cell value. At least three independent experiments were performed.

### 5-Ethynyl-2′-Deoxyuridine Assay

The cells were incubated with 5-ethynyl-2′-deoxyuridine (EdU; Ribobio) for 2 h and processed according to the manufacturer’s instruction. After three washes with phosphate-buffered saline (PBS), the cells were treated with 300 μl of 1× Apollo reaction cocktail for 10–30 min and analyzed by flow cytometry. For fluorescence microscope analysis, the EdU-stained cells in 96 wells were restained by DAPI (100 ng/ml) in the dark at room temperature for 10 min. After that, cells were washed with PBS twice and photographed on an ImageXpress Micro Confocal High-Content Imaging System (Molecular Devices, Sunnyvale, CA, United States).

### Annexin V/Propidium Iodide Assay (FACS Analysis for Differentiation of Apoptosis From Necrosis)

To analyze the extent of apoptosis and necrosis in response to SHK exposure, A549 and PC-9 cells in the exponential growth phase (2.5 × 10^5^ cells) were seeded in 12-well plate and were incubated at 37°C for the indicated times in the presence or absence of specified test drugs. Then the cells were trypsinized, pelleted, washed in ice-cold PBS, and resuspended in 1× binding buffer according to the manufacturer’s instructions. They were then incubated with annexin V–fluorescein isothiocyanate (FITC) and propidium iodide (PI) for 15 min at room temperature. After incubation, the stained cells were analyzed by flow cytometry (Cytoflex S, Beckman Coulter), and the emitted fluorescence of the FITC-stained cells exited by a 488-nm laser, whereas the emitted fluorescence of the PE-stained cells was measured at a wavelength of 561-nm laser. Cells were then gated by forward and side scattering, at least 10,000 events were used in calculations for each sample, and the proportions of viable, early apoptotic, late apoptotic, and necrotic cells in each sample were estimated from those with low annexin–low PI, high annexin–low PI, high annexin–high PI, and low annexin–high PI staining, respectively.

### RNA Extraction and Quantitative Real-Time Polymerase Chain Reaction

Total RNA from the cells was extracted using TRIzol (Invitrogen) according to the manufacturer’s protocol and then quantified using a NanoDrop spectrophotometer (Thermo Fisher Scientific, United States). One microgram of RNA was reversely transcribed into complementary DNA (cDNA) using PrimeScript reverse transcription (RT) reagent kit with genomic DNA Eraser (TaKaRa, China) and Bulge-Loop-miRNA–quantitative RT (qRT)–polymerase chain reaction (PCR) Starter Kit (RiboBio, China). qRT-PCR reactions were performed to detect miR-628-3p and p53 mRNA expression using SYBR Green I mix reagents (TaKaRa, China) in a 20-μl reaction volume (10 μl SYBR Green I mix, 200 nM forward and reverse primer, 1 μl cDNA template) on a 7500 Real-Time PCR System (Applied Biosystems). 18sRNA or U6 was used as the internal control. Each reaction was run in triplicate. The change in gene expression was calculated with the 2^−ΔΔCt^ method. The details of PCR primers are shown in Supplementary Table S2.

### Protein Extraction

Cells were washed three times with PBS chilled to 4°C. Whole-cell proteins were extracted with M-PER Mammalian Protein Extraction Reagent (78503, Thermo Fisher Scientific, United States) containing protease and phosphatase inhibitor (Roche, Germany) at 4°C for 30 min. Then, the samples were centrifuged at 14,000 × *g* for 10 min, and the supernatant was transferred to a new tube for analysis.

### Western Blotting

Before blotting, the protein was quantified using the bicinchoninic acid method. Simple Western immunoblotting was performed on a Simple Wes System (ProteinSimple, CA, United States) using a Size Separation Master Kit with Split Buffer (12–230 kDa) according to the manufacturer’s standard instruction and using anti-p53 (sc-126, Santa Cruz, USA) and anti–β-actin (4970S, CST, United States) antibodies. Compass software (version 4.0.0, ProteinSimple) was used to program the Simple Wes and for presentation (and quantification) of the Western immunoblots. Output data were displayed from the software calculated average of seven exposures (5–480 s).

### Luciferase Reporter Assays

The wild-type (WT) promoter region for the transcription of pre–miR-628 (2 kb upstream of the pre–miR-628 gene) was cloned into the pGL3 basic luciferase vector (Promega). Then, the mutant-type (MUT) promoter region that lost p53 binding sites was also synthesized and cloned into the pGL3 basic luciferase vector. A dual-luciferase reporter assay was carried out by cotransfecting the pGL3 basic luciferase vector containing the WT or MUT promoter region (see Supplementary Figure S2) of pre–miR-628, p53-expressing plasmid, empty vector control, and *Renilla* luciferase vector into 293T cells using the Lipofectamine 3000 reagent (Thermo Fisher Scientific). The firefly and *Renilla* luciferase activity were measured with the dual-luciferase reporter assay system (Promega). Firefly luciferase activity was normalized to *Renilla* activity and presented as relative luciferase activity. All assays were performed in triplicate three times.

### Chromatin Immunoprecipitation

Chromatin immunoprecipitation (ChIP) assays were performed using a Pierce Agarose ChIP Kit (Thermo Fisher Scientific) following the manufacturer’s instructions, and ChIP-enriched DNA samples were analyzed by qPCR. Cells were cross-linked with 1% formaldehyde for 10 min at room temperature and quenched in glycine. Anti-p53 antibody (CST #2524) or normal immunoglobulin G (IgG) (BD Biosciences) were used for immunoprecipitation. The DNA was recovered and subjected to qPCR to amplify the binding sites of the pre–miR-628 promoter region. Data are presented as relative enrichment normalized to control IgG. The primer pairs used for PCR analysis were as follows: forward, 5′-CAGTAGTTG​CCT​TGT​AAA​GTG​C-3′, reverse, 5′- AGA​AGA​GCG​AAA​ATG​ACA​GAC​C-3′;

### Statistical Analyses

Each experiment was performed at least three times, and all values are expressed as the mean ± SD of triplicate samples. Data are assessed by one-way analysis of variance with Tukey *post hoc* test. *p* < 0.05 was considered statistically significant.

## Results

### SHK Inhibits the Proliferation of A549 and PC-9 Cells

**FIGURE 1:**
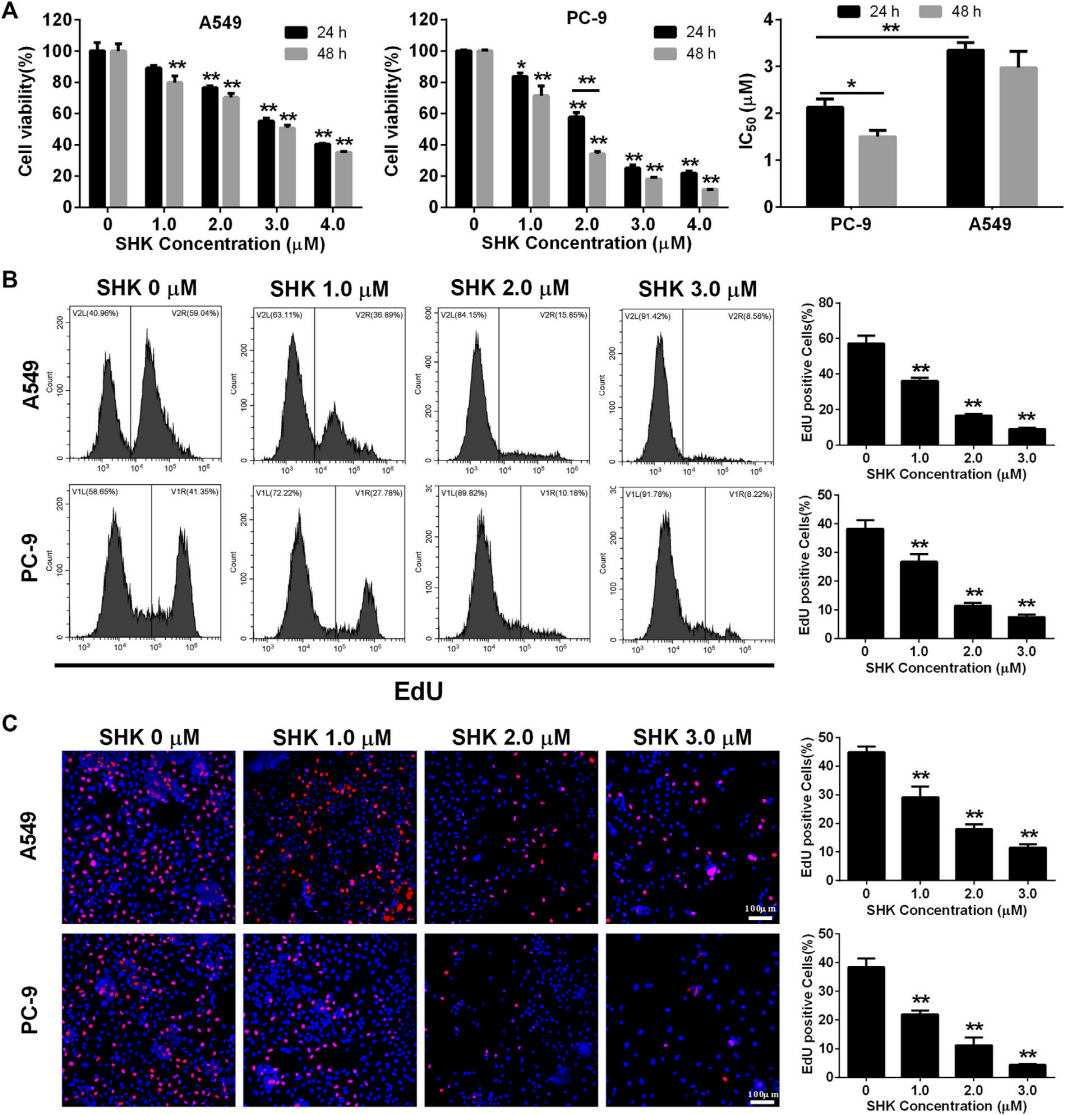
The inhibitory effect of SHK on A549 cells and PC-9 cells. **(A)** CCK-8 assay, **(B)** EdU assay on A549 (upper panel) and PC-9 **(lower panel)** cells detected by flow cytometry, and **(C)** EdU assay on A549 **(upper panel)** and PC-9 **(lower panel)** cells recorded by fluorescence microscope. All measurements were carried out in triplicates, and data are given as mean ± SD (*n* = 3). ^*^fnlowast *p* < 0.05, ^**^ *p* < 0.01.

A549 and PC-9, as a p53 WT cells (Raynal et al., 1997; Wang et al., 2015), were selected to investigate the role of SHK. Cell survival was measured with a CCK-8 assay. The results revealed a dose-dependent decrease in proliferation of A549 and PC-9 cells after SHK treatment, and the inhibition rate of concentration greater than 2.0 μM was significantly different from that of the untreated group (Figure 1A). In general, the inhibition rate of SHK on A549 and PC-9 cells treated for 48 h was higher than that of corresponding concentration treatment for 24 h, but there was no significant difference observed in both cell lines except the 2.0 μM treatment on PC-9 cells, so did the situation in IC_50_ concentration of SHK in A549 cells at 48 and 24 h, but the significant difference was detected on PC-9 cells. Both A549 and PC-9 cell lines showed high sensitivity to SHK, and the IC_50_ concentrations at 24 h were 3.349 ± 0.167 and 2.132 ± 0.174 μM, respectively.

EdU incorporation method, which can accurately reflect the percentage of cell proliferation (Yu et al., 2009), was also used to determine the proliferation inhibitory activity of SHK in A549 and PC-9 cells. The results of flow cytometry showed that 1.0 μM SHK treatment significantly reduced the proliferation of A549 and PC-9 cells; the EdU positive cells decreased by 40.14 and 34.98%, respectively (Figure 1B). While 3.0 μM SHK treatment almost completely blocked the proliferation of A549 and PC-9 cells (Figure 1B), which was reconfirmed by fluorescence microscope (Figure 1C). Moreover, the IC_50_ of SHK against A549 and PC-9 cells were 1.221 ± 0.059 and 1.334 ± 0.114 μM, respectively. Obviously, compared with CCK-8 results, EdU results showed that SHK had a stronger inhibitory effect and exhibited similar inhibitory activity in A549 and PC-9 cells.

### SHK Induces the Apoptosis of A549 and PC-9 cells

**FIGURE 2:**
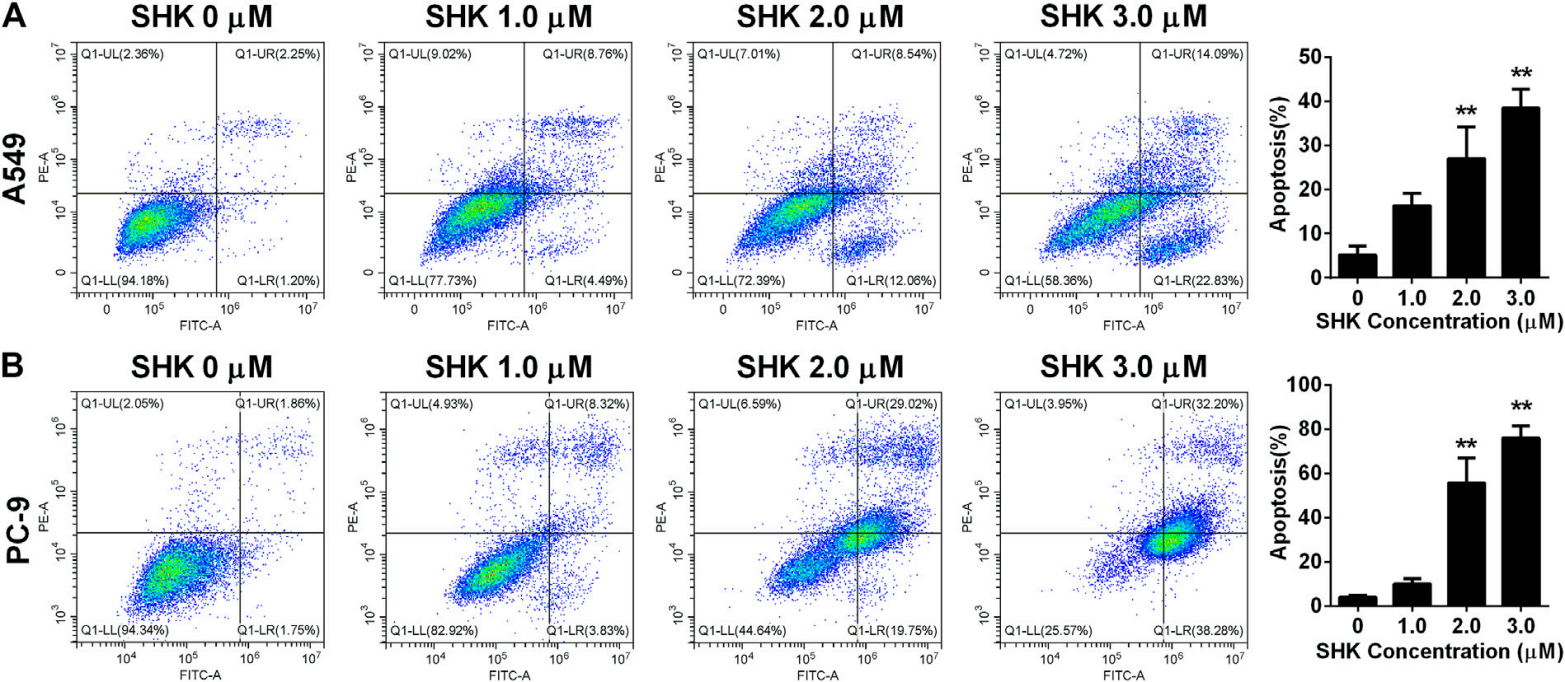
Apoptosis of **(A)** A549 cells and **(B)** PC-9 cells induced by SHK. A549 cells and PC-9 cells were treated by various concentrations of SHK for 24 h; cells were stained with annexin V–fluorescein isothiocyanate/propidium iodide and evaluated for apoptosis by flow cytometry. All measurements were carried out in triplicates, and data are given as mean ± SD (*n* = 3). ^**^ *p* < 0.01.

To further investigate whether SHK inhibited A549 and PC-9 cell growth through the induction of apoptosis, the percentage of apoptotic cells was calculated by flow cytometry using the annexin V/PI double-staining assay following treatment of the cells with various doses of SHK. The representative flow cytometry data are presented in Figure 2. It was demonstrated that treatment with 1.0, 2.0, and 3.0 μM SHK for 24 h significantly increased the numbers of apoptotic cells compared with the control group, and the numbers of apoptotic cells in both A549 and PC-9 cells increased in a dose-dependent manner. It is worth noting that, in treatment with 2.0 μM SHK, the apoptosis rates of A549 and PC-9 cells were 26.977 ± 7.127% and 55.787% ± 11.248%, respectively. The PC-9 cells showed more sensitivity to the apoptosis induced by SHK.

### Expression of p53 and miR-628-3p Changes in Response to SHK in A549 and PC-9 Cells

**FIGURE 3:**
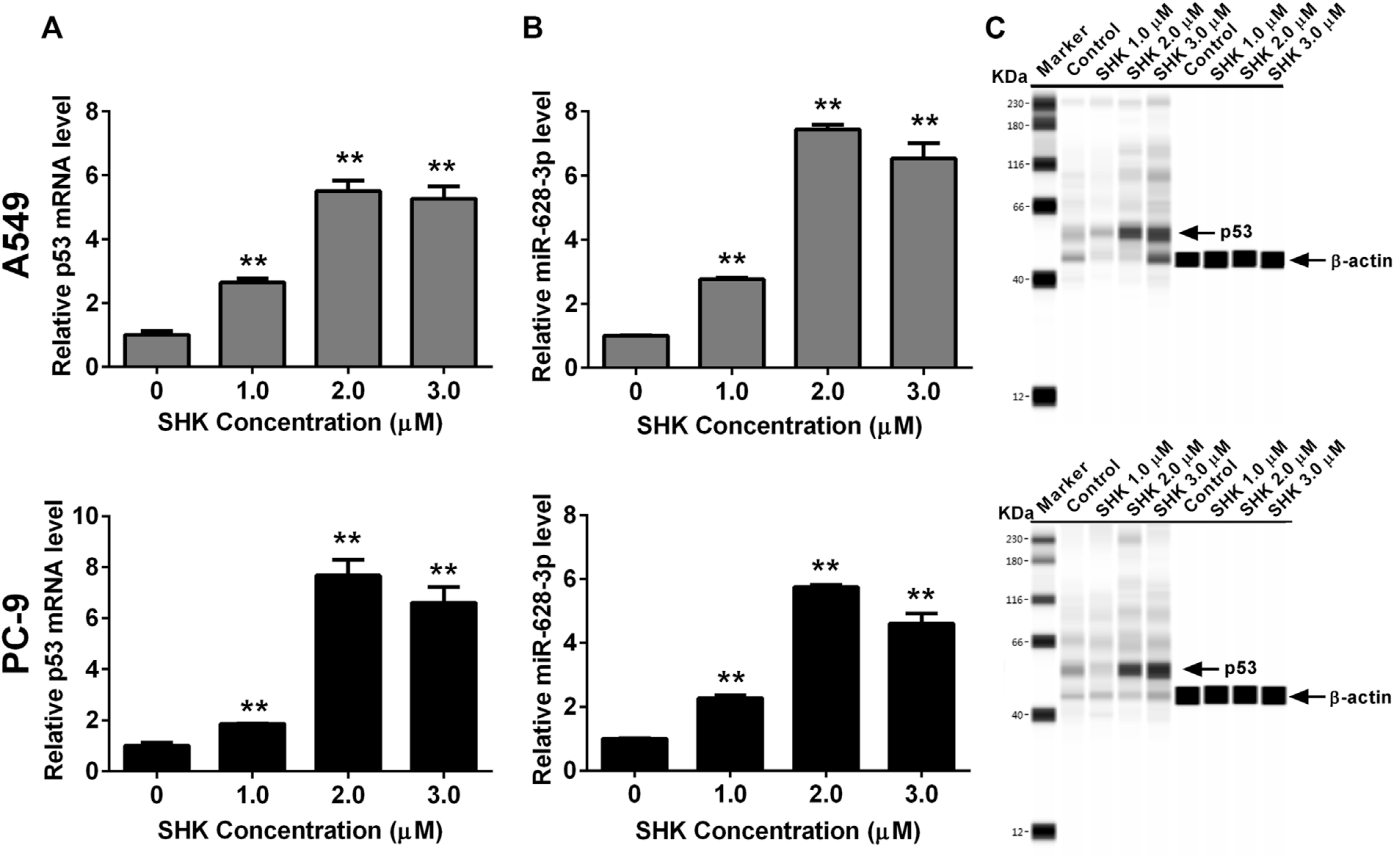
SHK induces the expression of p53 and miR-628-3p on A549 and PC-9 cells. Cells were treated with different concentrations of SHK for 24 h, and then the relative p53 **(A)** and **(B)** miR-628-3p mRNA levels were measured by qRT*-*PCR, and **(C)** relative p53 protein expression levels were recorded by simple Western immunoblotting method. All measurements were carried out in triplicates, and data are given as mean ± SD (*n* = 3). ^*^ *p* < 0.05, ^**^ *p* < 0.01.

Both p53 (Wang et al., 2013) and miR-628-3p (Pan et al., 2018) can mediate the apoptosis of A549 cells, and SHK can promote the apoptosis of A549 cells in a dose-dependent manner. Therefore, we studied whether p53 and miR-628-3p are involved in SHK-induced apoptosis of A549 and PC-9 cells. The results displayed that the relative miR-628-3p level and p53 mRNA and protein level were dramatically increased by SHK treatment compared with the control group (Figure 3). This positive reaction implied that SHK may exert its activity through up-regulating p53 and miR-628-3p.

### The Effects of miR-628-3p and p53 in A549 and PC-9 Cell Proliferation and Apoptosis

**FIGURE 4:**
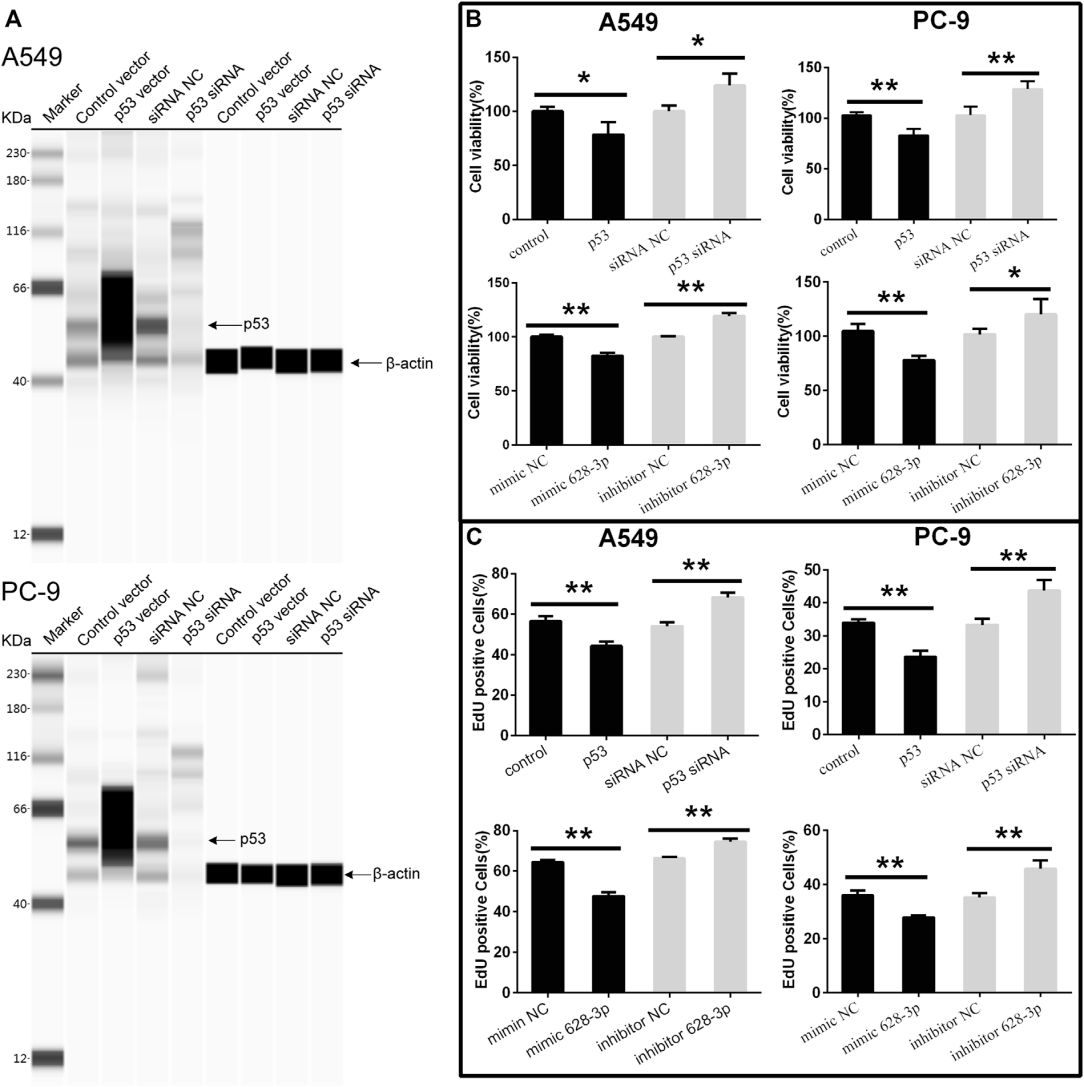
Cell viability of A549 and PC-9 cells. p53-overexpression plasmid (p53) or its control vector, p53 specific siRNA or siRNA NC, mimic 628-3p, inhibitor 628-3p, or their corresponding negative control was transfected into A549 or PC-9 cells by Lipofectamine 3000 as indicated. Then **(A)** total proteins were extracted, and p53 protein levels were determined on a Simple Wes System; **(B)** CCK-8 assay and **(C)** EdU assay were performed after 24 h of transfection. All measurements were carried out in triplicates, and data are given as mean ± SD (*n* = 3). ^*^ *p* < 0.05, ^**^ *p* < 0.01.

To understand the effects of p53 and miR-628-3p on the proliferation of A549 and PC-9 cells, p53 overexpression vector (or mimic 628-3p) and p53 siRNA (or inhibitor 628-3p) were transfected into A549 and PC-9 cells, respectively. Empty plasmid (mimic NC) and siRNA NC (inhibitor NC) were used as negative control respectively. The results showed that overexpression of p53 significantly elevated the protein level of p53 in A549 and PC-9 cells (Figure 4A) and led to the growth of A549, and PC-9 cells were inhibited (Figure 4B), but after interfering with siRNA of p53, the protein level of p53 was dramatically decreased (Figure 4A), and accordingly, the proliferation of A549 and PC-9 cells was promoted instead (Figure 4B). Similarly, the overexpression of mimic 628-3p also inhibited the growth of A549 and PC-9 cells, while the transfection of miR-628-3p inhibitor significantly increased the cell proliferation (Figure 4B). The effects of p53 and miR-628-3p on the proliferation of A549 and PC-9 cells were further reconfirmed using EdU assay (Figure 4C).

**FIGURE 5:**
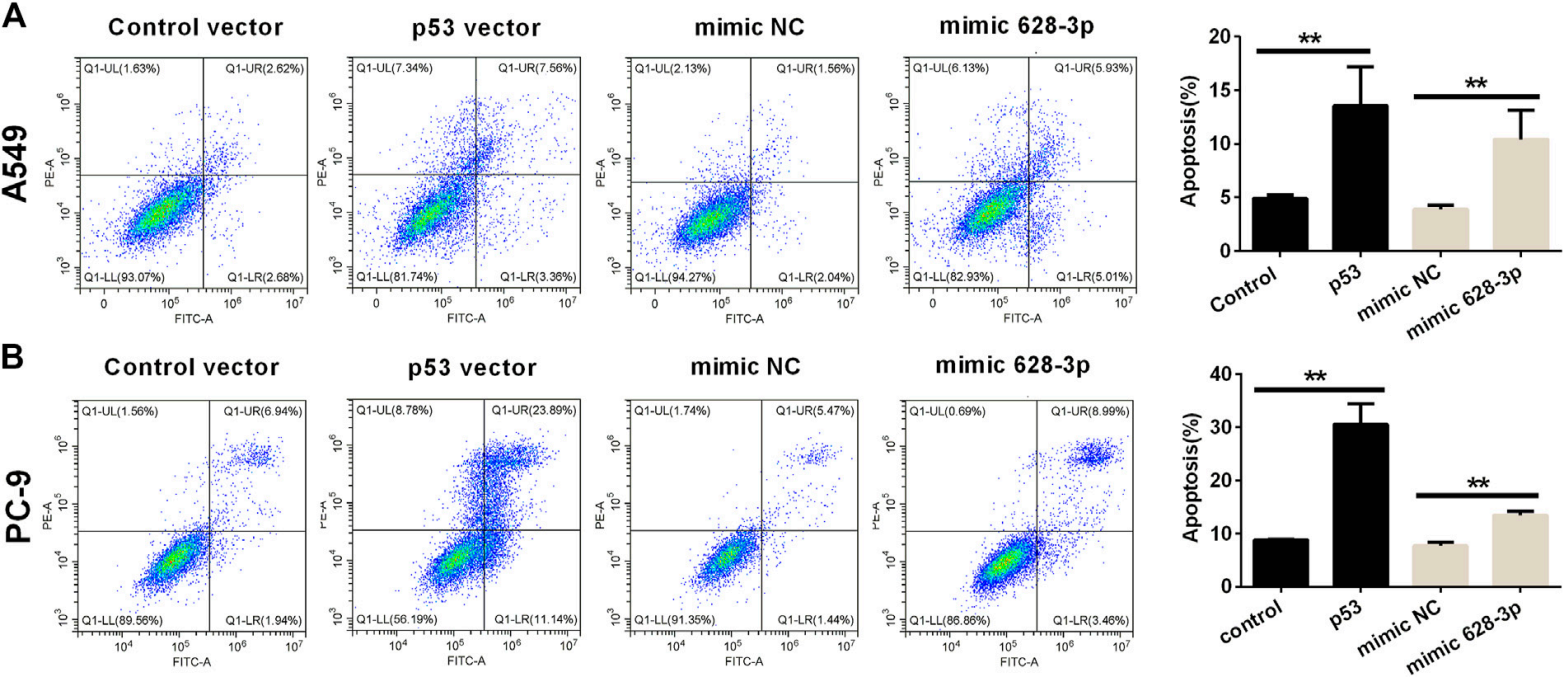
Effects of overexpression of miR-628-3p and p53 on apoptosis of A549 and PC-9 cells. **(A)** A549 and **(B)** PC-9 cells were transfected with p53 vector or control vector or mimic 628-3p or mimic NC, and the apoptosis was detected by flow cytometry 24 h later. All measurements were carried out in triplicates, and data are given as mean ± SD (*n* = 3). ^**^ *p* < 0.01, comparing with control vector or mimic NC.

The apoptosis of A549 cells induced by overexpression of miR-628-3p and p53 was analyzed by flow cytometry. The overexpression of p53 remarkably increased the apoptotic rates in A549 and PC-9 cells compared with control vector (Figure 5). Similarly, after transfection of mimic 628-3p, the apoptosis rate of A549 and PC-9 cells increased from 3.917% ± 0.361% to 10.430% ± 2.721% and from 7.710 ± 1.192% to 13.497% ± 1.222%, respectively (Figure 5).

### miR-628-3p is a Direct Transcriptional Target of p53

**FIGURE 6:**
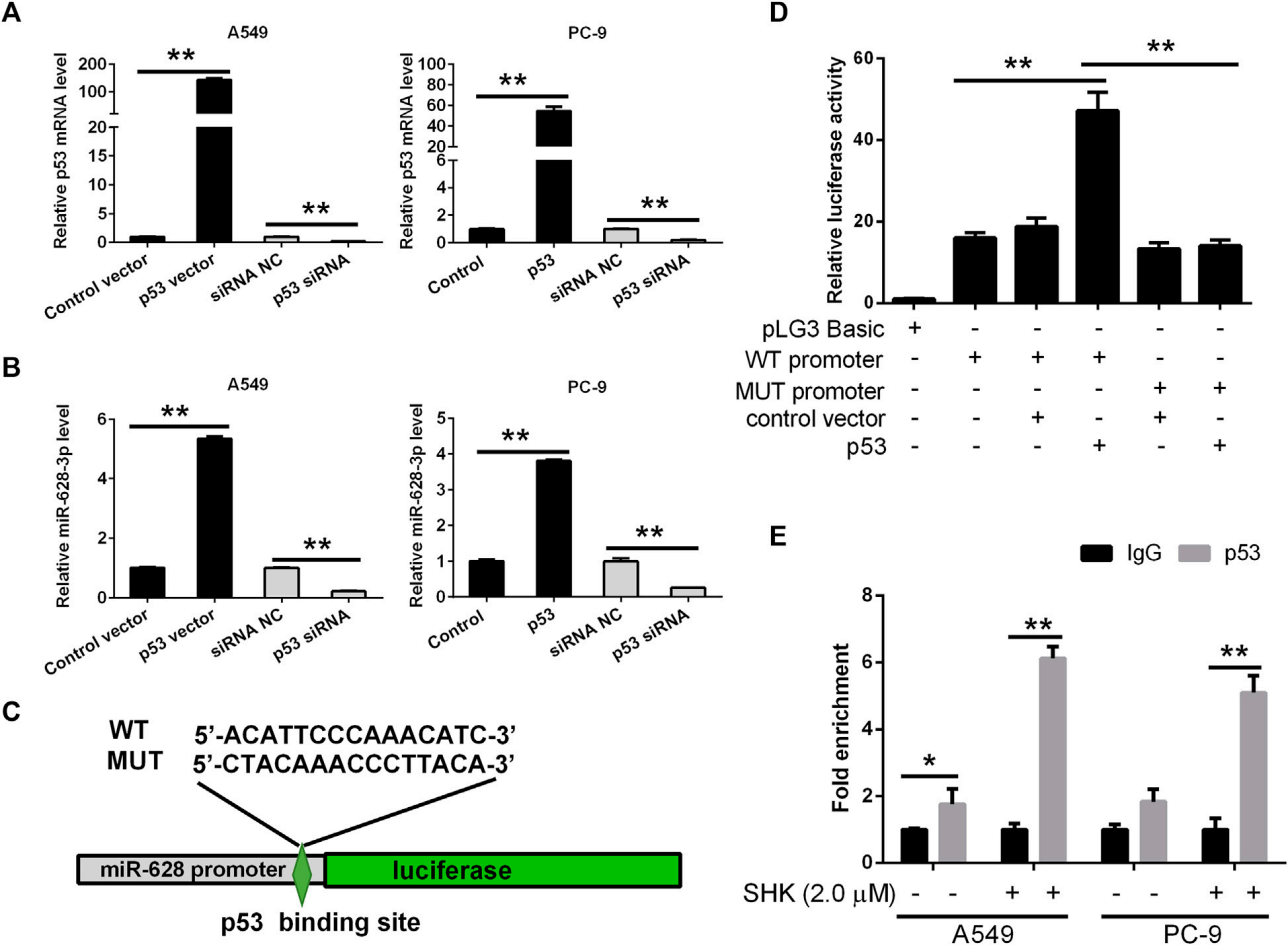
miR-628-3p is a direct transcriptional target of p53. After 24 h of p53 overexpression or interference, the expression levels of **(A)** p53 mRNA and **(B)** miR-628-3p were detected by qRT-PCR, respectively; **(C)** miR-628 reporter plasmid in which the pre–miR-628 promoter, either wild type or mutagenized in the p53 binding sites (depicted schematically), was cloned into pGL3 vector upstream of the luciferase reporter gene. **(D)** pGL3 luc reporter plasmid itself (pGL3 Basic) or pGL3 plasmid containing the WT or MUT promoter region of pre–miR-628, p53-expressing plasmid (p53), or its control vector was transfected into 293T cells by Lipofectamine 3000 as indicated. The interaction between p53 and the promoter region of pre–miR-628 was then determined by dual-luciferase assays. **(E)** A549 and PC-9 cells were untreated or treated with 2.0 μM SHK; after cell lysis, the chromosomal DNA was subjected to ChIP assays by incubating with mouse anti-human p53 antibody or normal mouse IgG. The DNA was recovered and subjected to qPCR to amplify the binding sites of the pre–miR-628 promoter region using specific primers. Data are presented as relative enrichment normalized to control IgG. All measurements were carried out in triplicates, and data are given as mean ± SD (*n* = 3). ^*^ *p* ≤ 0.05, ^**^ *p* ≤ 0.01.

Based on the data accumulated, we sought to investigate in detail the p53/miR-628-3p interplay. In keeping with the *in silico* predictions of a p53-mediated control of miR-628-3p, we observed that modulation of p53 expression in tumor cell lines affected miR-628-3p transcription. Specifically, ectopic expression of p53 or siRNA mediated down-regulation of p53 in A549 and PC-9 cells associated with a concordant variation in the expression of miR-628-3p (Figures 6A, B), which implied that p53 is a regulator of miR-628-3p.

To validate this hypothesis, the 2-kb promoter sequence of human pre–miR-628 was retrieved from UCSC database, and the binding of transcription factor p53 to the promoter sequence was predicted by Jaspar. Then we generated an miR-628-3p promoter reporter plasmid in which the miR-628-3p promoter, either WT or mutagenized in the p53 binding sites with the highest prediction score according to MatInspector, was cloned upstream of the luciferase gene (Figure 6C). Reporter assays indicated that cotransfection of p53 and miR-628-3p WT promoter reporter luc plasmid dramatically increased the luciferase activity compared with the control group, whereas cotransfection of p53 and miR-628-3p MUT promoter reporter luc plasmid hardly detected the increase of luciferase activity (Figure 6D).

To ultimately demonstrate that p53 actually sits on the miR-628-3p promoter, we performed ChIP experiments. A549 and PC-9 cells lysates were immunoprecipitated using a p53-specific antibody and the regions encompassing the pre–miR-628 promoter was amplified and quantified by qPCR. Preimmune IgG isotype antibodies were used in a mock immunoprecipitation as a negative control/background signal. ChIP-qPCR confirmed that there was a certain amount of p53 binding on the promoter of pre–miR-628 of A549 cells without any treatment, whereas a strong enrichment of the amplicons encompassing was observed in anti-p53 ChIP compared to the control IgG ChIP after stimulation with 2.0 μM of SHK on both A549 and PC-9 cells (Figure 6E). The identification of functional binding sites for p53 in the pre–miR-628 promoter region compellingly demonstrated that miR-628-3p is a direct transcriptional target of p53.

### SHK Inhibits A549 and PC-9 Cell Proliferation and Induces A549 and PC-9 Cell Apoptosis Through the p53/miR-628-3p Axis

**FIGURE 7:**
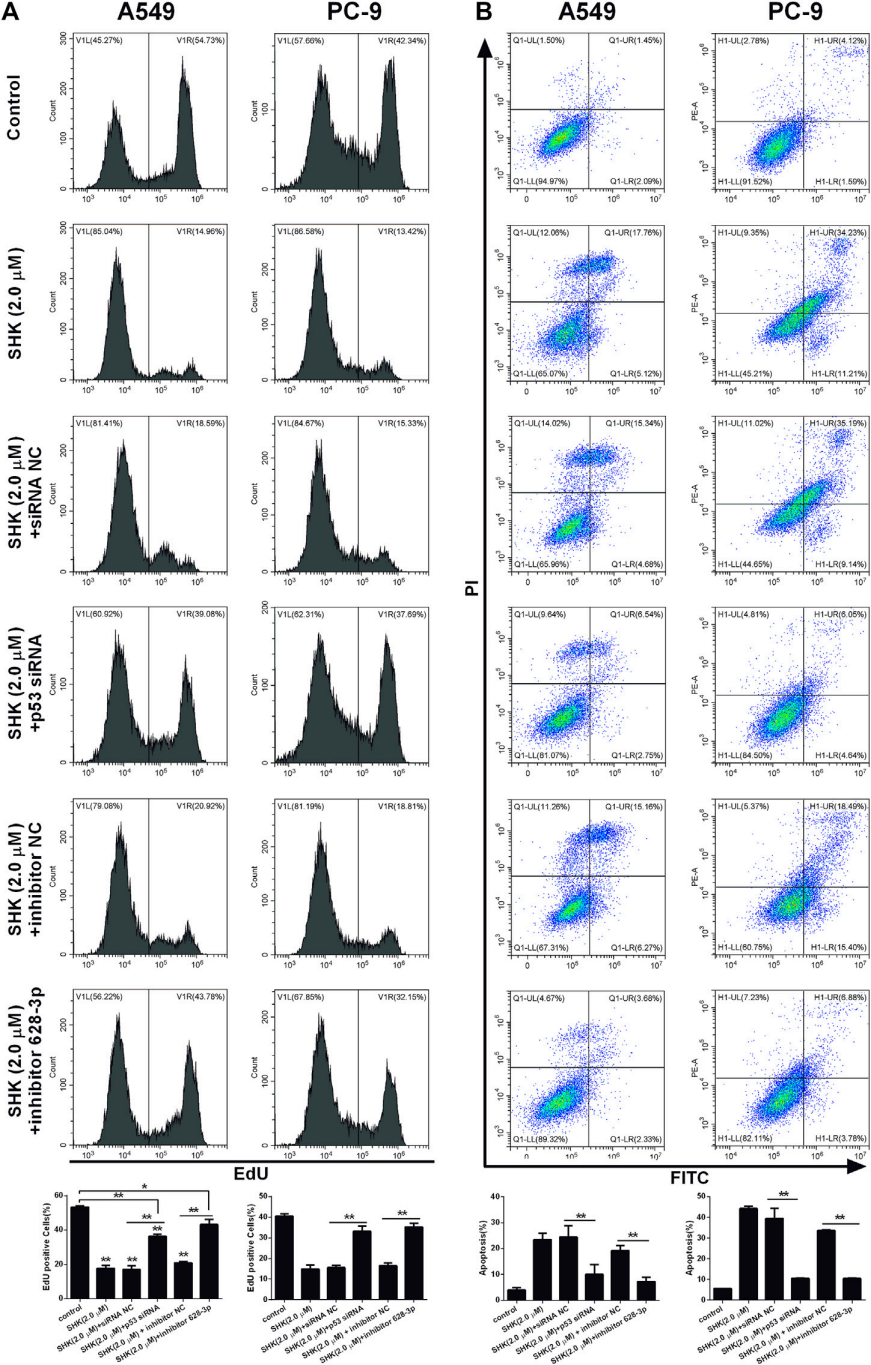
SHK inhibits the growth and induces apoptosis of A549 and PC-9 cells through the p53/miR-628-3p pathway. A549 and PC-9 cells were pretreated with p53 siRNA or inhibitor 628-3p and then cotreated with 2.0 μM of SHK for 24 h; cells were stained with **(A)** EdU or **(B)** annexin V–fluorescein isothiocyanate/propidium iodide and analyzed by flow cytometry. siRNA NC and inhibitor NC were performed as negative control, respectively. All measurements were carried out in triplicates, and data are given as mean ± SD (*n* = 3). ^*^ *p* ≤ 0.05, ^**^ *p* ≤ 0.01.

We investigated if SHK could be able to inhibit the proliferation of A549 and PC-9 cells through p53/miR-628-3p pathway, EdU assay, which could accurately detect cell proliferation was conducted by flow cytometry. As depicted in Figure 7A, the inhibitory effects of SHK (2.0 μM) on the proliferation of A549 and PC-9 cells were significantly attenuated by addition of p53 specific siRNA or miR-628-3p–specific inhibitor. Accordingly, the apoptosis of A549 and PC-9 cells induced by SHK (2.0 μM) was also dramatically abolished by cotreatment with p53 siRNA or inhibitor 628-3p (Figure 7B). Overall, these data added support to the notion that SHK inhibits A549 and PC-9 cell proliferation and induces A549 and PC-9 cell apoptosis through up-regulation p53/miR-628-3p signal pathway.

## Discussion

SHK, a naphthoquinone derivative, has been shown to inhibit the growth of many kinds of cancer cells. Studies have shown that lung cancer cells are more sensitive to SHK than other types of cancer cells. For example, the IC_50_ values of SHK on MCF-7, HeLa, and HepG2 cells were 5.0, 5.8, and 9.4 μM respectively, but its IC_50_ against A549 cells ranging from 1.7 to 3.1 μM (Wei et al., 2020), which was almost consistent with our results 3.349 and 2.132 μM against A549 and PC-9 cells determined by CCK-8 method, respectively (Figure 1A). EdU, a thymidine analogue, can be incorporated into DNA during its synthesis, making the counting of proliferating cells visible, simple and more accurate (Okada and Shi, 2017). The result of EdU assay indicated that the IC_50_ of SHK on A549 and PC-9 cells were 1.221 and 1.334 μM, respectively (Figure 1B), which reconfirmed the strong inhibition activity of SHK against NSCLC. The apoptosis of A549 and PC-9 cells induced by SHK was analyzed by flow cytometry. The results revealed that SHK could induce the apoptosis of A549 and PC-9 cells in a dose-dependent manner, and the apoptosis rates were 21.547% ± 3.731% and 47.787% ± 2.674%, respectively, when treated with 2.0 μM of SHK (Figure 2). These data warrant additional research to further investigate the inhibitory effect of SHK against A549 and PC-9 cells and the biochemical mechanisms behind its bioactivities.

SHK can regulate a variety of signaling pathways and thus exert its inhibitory activity on tumor cells (Eric et al., 2020). Our previous study found that miR-628-3p can regulate the proliferation and apoptosis of A549 cells (Pan et al., 2018). Bioinformatics analysis showed that the promoter sequence of pre–miR-628 contained p53 binding sites, which suggested that p53 might be an miR-628-3p regulatory factor. In view of the important regulatory role of p53 on cell proliferation and apoptosis, we speculate that SHK may regulate miR-628-3p through p53 to mediate the proliferation inhibition and apoptosis induction of A549 and PC-9 cells. The results showed that SHK could simultaneously promote the expression of p53 and miR-628-3p in a dose-dependent manner (Figure 3). Overexpression of p53 or miR-628-3p can inhibit the proliferation and induce apoptosis of A549 and PC-9 cells. Meanwhile, interference with p53 or inhibition of miR-628-3p has the opposite effect (Figures 4, 5). In addition, there was overexpression of p53 or interference with p53 associated with a synergistic change in the expression of miR-628-3p (Figures 6A, B). These data reconfirmed the inhibitory and proapoptotic effects of p53 (Zhang et al., 2019) and miR-628-3p (Pan et al., 2018) on A549 and PC-9 cells and further suggested that p53 may regulate the transcription of miR-628-3p, which may be a new anticancer mechanism of SHK.

To further verify the interaction between p53 and miR-628-3p, we performed a dual luciferase reporting assay. The results displayed that p53 could interact with the promoter region of pre–miR-628, leading to the elevation of relative luciferase activity from 18.769 ± 3.648 to 47.180 ± 7.995, whereas mutation of the binding site completely abolished this interaction and as indicated by no luciferase activity promoted (Figure 6D). Moreover, we applied a ChIP assay, the standard method to determine the interaction between specific proteins and their DNA targets *in vivo* (Isono and Hashimoto, 2020), to assess the direct interaction between p53 and the promoter of pre–miR-628. Consistently, a direct interaction was detected and was weak in control cells; however, the interaction was dramatically enhanced after SHK (2.0 μM) treatment (Figure 6E). As Western blot results showed that SHK could promote the expression of p53 in a dose-dependent manner (Figure 3C); therefore, these data suggested that p53 is a regulator of miR-628-3p, and SHK can promote the expression of miR-628-3p by up-regulating the expression of p53. Finally, we performed gene knockdown for p53 or miR-628-3p to further verify the possible mechanism of SHK against A549 and PC-9 cells. The results clearly demonstrated that p53 or miR-628-3p silencing robustly decreased inhibiting cell proliferation activity and inducing cell apoptosis activity of SHK (Figure 7).

**FIGURE 8:**
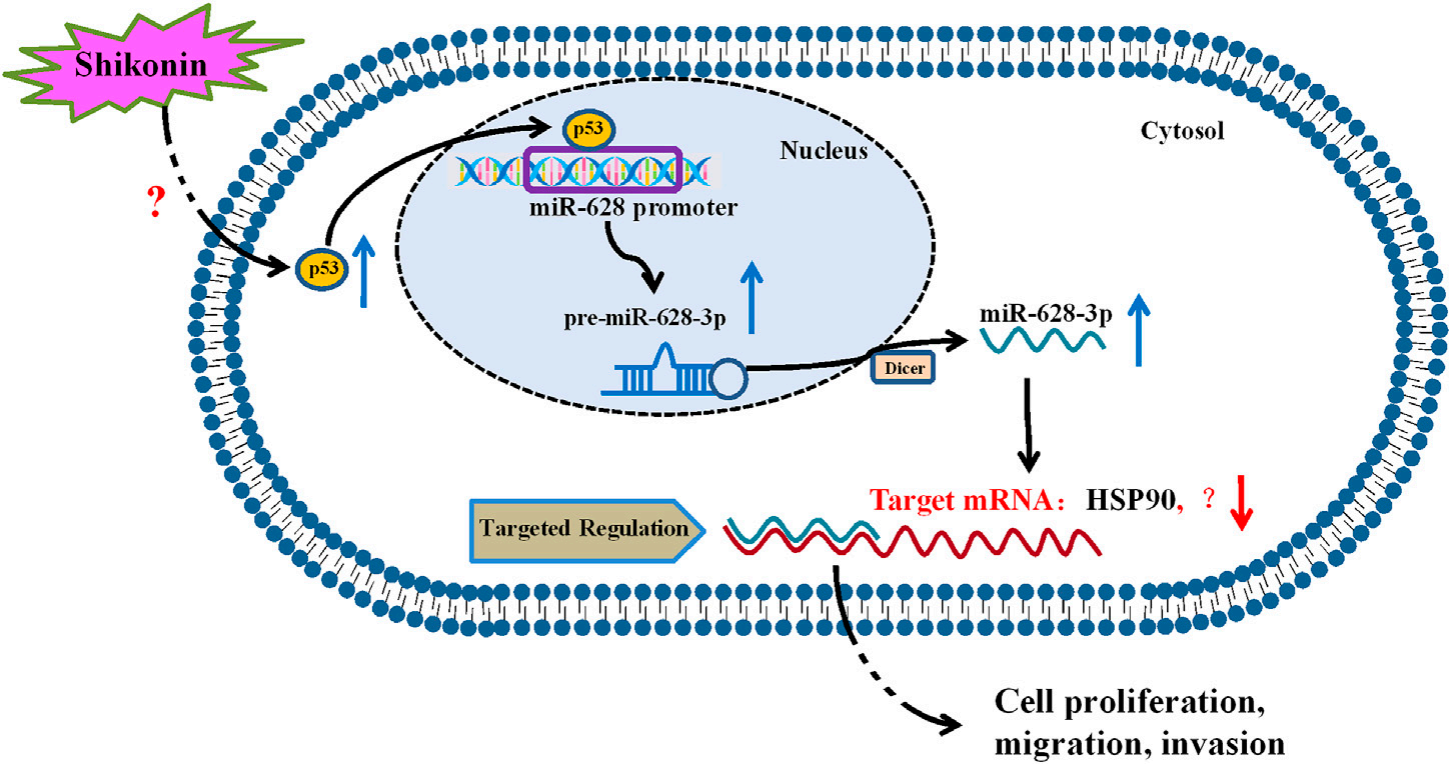
Proposed mechanism of SHK inhibiting cell growth and inducing cell apoptosis in non–small cell lung cancer by regulating p53/miR-628-3p pathway.

In summary, our study revealed that p53 is a regulator of miR-628-3p, and elevated p53 could inhibit the growth and induce apoptosis of A549 and PC-9 cells by promoting miR-628-3p expression (Figure 8). In addition, we found that SHK inhibits the growth and induces apoptosis of A549 and PC-9 cells at least partly by up-regulating p53/miR-628-3p signaling pathway (Figure 8). Moreover, our previous work displayed that HSP90 is one of the downstream targets of miR-628-3p (Pan et al., 2018), which implied that SHK may exert its effect through p53/miR-628-3p/HSP90 pathway, and more work is needed to uncover other potential targets (Figure 8). Many studies have confirmed that p53 is the key signal pathway for SHK to exert antitumor effect (Yeh et al., 2015; Zheng, 2017; Zheng et al., 2018), but the specific upstream target of direct interaction with SHK is still unclear (Figure 8). Affinity-based protein profiling would be a powerful technique to reveal the exact target protein of SHK (van der Zouwen and Witte, 2021). Systematically revealing the target and molecular mechanism of SHK is conducive to promoting its development and utilization and looking for new high-efficiency anticancer drugs. Therefore, these novel findings provide an alternative approach to target p53/miR-628-3p axis and could be used for the development of new treatment strategies for NSCLC.

## Data Availability

The original contributions presented in the study are included in the article/Supplementary Material, further inquiries can be directed to the corresponding author.
