# Supplementary material for: Up-Regulation of p53/miR-628-3p Pathway, a Novel Mechanism of Shikonin on Inhibiting Proliferation and Inducing Apoptosis of A549 and PC-9 Non–Small Cell Lung Cancer Cell Lines
Source: Front Pharmacol. 2021 Nov 16;12:766165. doi: 10.3389/fphar.2021.766165 (PMC8635033; doi:10.3389/fphar.2021.766165)
Supplement: Supplementary file 1 [file Table1.DOCX]

Supplementary Table 1 Catalogue number or Sequences of miRNA mimics, miRNA inhibitors and si-RNAs.

| Product Name | Catalogue number or Sequences | Company |
| --- | --- | --- |
| miR-628-3p mimic | miR10003297-1-5 | Ribobio, Guangzhou |
| mimic NC | miR1N0000001-1-5 | Ribobio, Guangzhou |
| miR-628-3p inhibitor | miR20003297-1-5 | Ribobio, Guangzhou |
| inhibitor NC | miR2N0000001-1-5 | Ribobio, Guangzhou |
| siR NC | siN0000001-1-5 | Ribobio, Guangzhou |
| si-p53 | GGAGTATTTGGATGACAGA | Ribobio, Guangzhou |
