# Supplementary material for: Up-Regulation of p53/miR-628-3p Pathway, a Novel Mechanism of Shikonin on Inhibiting Proliferation and Inducing Apoptosis of A549 and PC-9 Non–Small Cell Lung Cancer Cell Lines
Source: Front Pharmacol. 2021 Nov 16;12:766165. doi: 10.3389/fphar.2021.766165 (PMC8635033; doi:10.3389/fphar.2021.766165)
Supplement: Supplementary file 4 [file Table2.DOCX]

Supplementary Table 2 Catalogue number or sequences of primers.

| Name | | | Catalogue number or Sequences | Company |
| --- | --- | --- | --- | --- |
| p53 | forward | 5’-TTGAGGTGCGTGTTTGTG-3’ | | Sangon Biotech, Shanghai |
|  | reverse | 5’-CTGGGCATCCTTGAGTTC-3’ | |  |
| ChIP-qPCR | forward | 5’-CAGTAGTTGCCTTGTAAAGTGC-3’ | | Sangon Biotech, Shanghai |
|  | reverse | 5’- AGAAGAGCGAAAATGACAGACC-3’ | |  |
| 18sRNA | forward | 5’-TTGACTCAACACGGGAAACCT-3’ | | Sangon Biotech, Shanghai |
|  | reverse | 5’-AGAAAGAGCTATCAATCTGTCAATCCT-3’ | |  |
| miR-628-3p |  | MQPS0001997-1-100 | | Ribobio, Guangzhou |
| U6 |  | MQPS0000002-1-100 | | Ribobio, Guangzhou |
